# Novel nucleic acid origami structures and conventional molecular beacon–based platforms: a comparison in biosensing applications

**DOI:** 10.1007/s00216-021-03309-4

**Published:** 2021-04-06

**Authors:** Noemi Bellassai, Roberta D’Agata, Giuseppe Spoto

**Affiliations:** 1 Dipartimento di Scienze Chimiche, Università degli Studi di Catania, Viale Andrea Doria 6, 95125 Catania, Italy; 2 Consorzio Interuniversitario “Istituto Nazionale Biostrutture e Biosistemi”, c/o Dipartimento di Scienze Chimiche, Università degli Studi di Catania, Viale Andrea Doria 6, 95125 Catania, Italy

**Keywords:** DNA, Biosensor, Origami, Molecular beacon, Nanostructures, Fluorescence

## Abstract

Nucleic acid nanotechnology designs and develops synthetic nucleic acid strands to fabricate nanosized functional systems. Structural properties and the conformational polymorphism of nucleic acid sequences are inherent characteristics that make nucleic acid nanostructures attractive systems in biosensing. This review critically discusses recent advances in biosensing derived from molecular beacon and DNA origami structures. Molecular beacons belong to a conventional class of nucleic acid structures used in biosensing, whereas DNA origami nanostructures are fabricated by fully exploiting possibilities offered by nucleic acid nanotechnology. We present nucleic acid scaffolds divided into conventional hairpin molecular beacons and DNA origami, and discuss some relevant examples by focusing on peculiar aspects exploited in biosensing applications. We also critically evaluate analytical uses of the synthetic nucleic acid structures in biosensing to point out similarities and differences between traditional hairpin nucleic acid sequences and DNA origami.

**Graphical abstract:**

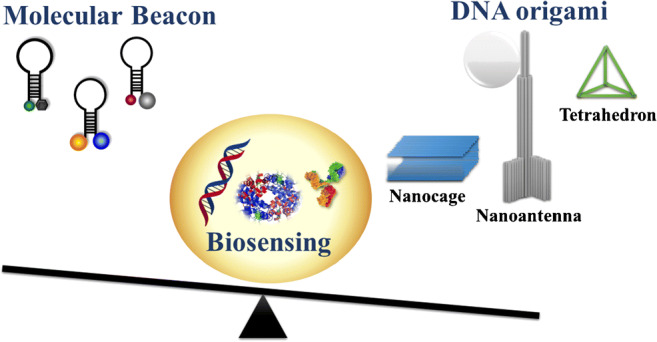

## Introduction

Nucleic acid (NA) nanotechnology designs and develops synthetic nucleic acid strands to fabricate nanosized functional systems. Such systems are exploited in biosensing [1–5] and computing fields [6, 7], molecular transport processes [8–10], and catalysis [11, 12]. Structural properties and the conformational polymorphism of nucleic acid sequences are inherent characteristics that make them attractive biomaterials in biosensing. The defined NA sequence allows for knowing the DNA scaffolds’ possible interactions and thermodynamics. Such a peculiar property can be easily exploited to design spatially controlled nanostructures suitable for preferential binding of a specific target compound [13–15].

Compared to other biomolecules used in biosensing, such as enzymes and antibodies, NA nanostructures exhibit improved stability [16]. Moreover, NA sequences can bind target molecules with good affinity and specificity, establishing various interactions, including hydrophobic and electrostatic interactions, hydrogen bonds, and covalent bonds. The most commonly adopted NA-based biosensing approach exploits synthetic single-stranded (ss) DNA recognition elements to detect the complementary sequence in a DNA target molecule through Watson-Crick base pairs [17]. A similar approach is adopted to recognize selected nucleic acid sequences [18, 19] and detect single-base mismatches related to specific diseases [20]. Besides a facile design, DNA-based platforms’ main advantage is their specificity in identifying the target sequence by reporting significant discrimination in the detected signal. However, such platforms are limited to biosensing applications involving a small NA target family. For this reason, functional DNAs such as aptamers have also been designed and applied in the biosensor field, thanks to their particular recognition ability and biocompatibility with the cellular environment [21].

DNA aptamers are synthetic oligonucleotides able to detect different target molecules (including metal ions, organic dyes, proteins, amino acids, and even whole cells) with a good affinity (nanomolar to micromolar) and specificity by folding into secondary and tertiary structures. Aptamers are identified with a combinatorial method called systematic evolution of ligands by exponential enrichment (SELEX) because their function cannot be simply designed based on the primary structure predictable interactions [22]. Since aptamers have been first described, they have been widely used to build biosensors, leading to the evolution of DNA sensing performances [23, 24]. Furthermore, some DNA analogues, like peptide nucleic acids (PNA) and locked nucleic acids (LNA), demonstrated to be functional tools for high-performance affinity biosensors [25, 26]. Besides, circular NA moieties or backbone-modified (e.g., 2-O′-methyl) NAs have been used to prevent degradation by nucleases with no alteration of NA-based sensor functionalities [27].

The availability of various strategies for the functionalization of NA scaffolds has made it extremely advantageous to use such synthetic sequences as recognition elements and signal probes or as probe linkers/amplifiers. When employed in amplification methods such as hybridization chain reaction and catalytic hairpin assembly as strand-mediated signal amplifiers, NA scaffolds specifically hybridize the target molecule, thus triggering DNA amplicons’ continuous production. The cascade event responsible for the amplification is specific and allows achieving exceptional detection limits [28]. Such amplification methods can use functionalized NA scaffolds to generate fluorescence, electrochemical, magnetic, and electrochemiluminescence signals [29].

Several reactive groups operating as signalling moieties (i.e., fluorophore/quencher pairs or electrochemical redox labels) or anchoring tags (i.e., thiol, amino, and biotin groups) can be easily added to the DNA sequence to identify the specific interaction with the target molecule in sensing applications. In particular, when a DNA sequence is functionalized with fluorophore/quencher terminal groups, its shape transitions caused by the target hybridization can increase the distance between a tagged fluorophore and another fluorophore or a quencher, leading to alterations in the Förster resonance energy transfer (FRET) properties and, consequently, in the produced optical signal. Hairpin-shaped molecular beacon (MB) probes operate based on the above-described mechanism. The MB recognition of a NA target causes the linearization of the hairpin structure and the detection of the hybridization event by fluorophore/quencher pair [30]. Along this way, the use of different fluorescent or electroactive labelling probes to get different signal read-outs from different targets could be an appealing approach to achieve high-throughput multiplex detection.

NA nanotechnology has contributed to improving biosensor design and performance by exploiting the conformational polymorphism of DNA sequences, through the self-assembly of multiple NA fragments to fabricate innovative scaffolds called DNA origami [31]. DNA origami can be precisely assembled to build a wide range of flexible NA scaffolds in two-dimensional (2D) or three-dimensional (3D) frameworks taking advantage of hybridization reactions’ programmability. Some origami scaffolds are highly versatile and capable of achieving either extracellular or intracellular environments [32], where the sensing mechanism exclusively depends on the conformational change of the probe portion involved in target interaction. Probes, which can include aptamers or i-motifs (i.e., C-rich regions with intercalated parallel duplexes) connected to fluorophores and quenchers, are also applied for the recognition of small molecules or ions (e.g., ATP, Hg^2+^ H^+^) [33].

The technical aspects related to the design, assembly, and characterization of DNA origami structures have been already discussed in excellent reviews [34–36] and are not the focus of this review. Here, we review advances in DNA structures for biosensing applications over the last 5 years. We briefly present relevant DNA scaffolds divided into conventional hairpin MBs and DNA origami and discuss some relevant examples by focusing on peculiarities exploited in biosensing applications. We selected MB and origami scaffolds from those structures based on DNA sequences to provide a direct comparison in biosensing between the simple hairpin shape of MBs and the complex, multidimensional design of origami. Both systems exploit the same building block (DNA) and offer the possibility to precisely design the final structure’s geometry and function based on Watson-Crick base pairs. For those reasons, functional DNAs such as aptamers and DNAzymes are not reviewed together with DNA mimics such as PNA and LNA, whose building blocks are different than DNA building blocks. In the following section, we critically evaluate the practical analytical uses of synthetic DNA structures as recognition elements and probe signalling in biosensing to point out similarities and differences between traditional hairpin DNA sequences or MBs and DNA origami scaffolds. We highlight each of them’ pros and cons by also providing examples of applications exploiting their peculiar features. In light of these aspects, we finally provide future perspectives on these DNA-based structures in biosensing applications.

## Molecular beacon design and mechanisms

**Fig. 1:**
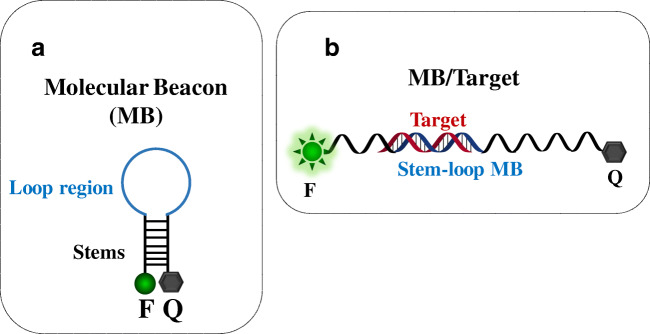
(A) Molecular beacon structure (MB) and (B) conformational change of the structure after the binding with a target sequence. F, fluorophore (donor dye); Q, quencher (acceptor dye)

In 1996, Tyagi and Kramer reported MB technology’s first application for single-strand DNA detection [37]. MB is based on a hairpin-shaped stem-loop structure and usually consists of two complementary stems bearing a donor dye (commonly referred to as the fluorophore, F) and an acceptor dye (the quencher, Q) at the two ends, respectively (Fig. 1A). The loop region is usually complementary to the target sequence. In its stable stem-loop configuration (close-state conformation) (Fig. 1A), MB produces a very low background fluorescence due to the fluorophore and quencher proximity. The interaction of the target molecule with the loop region’s complementary sequence (Fig. 1B) causes a conformational change of the hairpin structure (open-state conformation). The fluorophore and the quencher are then spatially separated, and an intense fluorescence is produced. MB probes can be described as sensing molecular systems able to switch between two different signalling conformations (close-state and open-state) according to the specific analyte’s presence and amount. For this reason, MB probes are used to detect and quantify nucleic acids and applied in clinical diagnosis, genotyping, and allele identification [37–41].

MBs’ selectivity depends on the stem sequences designed to ensure interactions keeping a stable close-state arrangement for MB and favour the conformational change after the hybridization with the target. Instead, MBs’ sensitivity depends on the signalling pair (fluorophore and quencher) attached to stems end termini. A fluorescent signal produced in the close-state conformation and/or a low-intensity signal generated in the open-state conformation translate into a reduced detection sensitivity.

Criteria for achieving optimal stability for MBs include its length, usually ranging between 25 to 35 nucleotides, sequence, guanine-cytosine (GC) content of both the stem and loop sequence, and the melting temperature of the MB-target duplex. The loop should include a 15 to 30 single-stranded sequence region complementary to the target sequence. The stem should have a melting temperature 7–10 °C higher than the detection temperature, and its sequence should include 5–7 bps [42].

Various signalling labels are available to obtain MBs producing high signal-to-background ratios. These include inorganic materials, organic compounds [43], nanomaterials, metal complexes, conjugated polymers, superquenchers (SQs), and other materials exhibiting superior photophysical properties for sensing purpose [43–45].

**Fig. 2:**
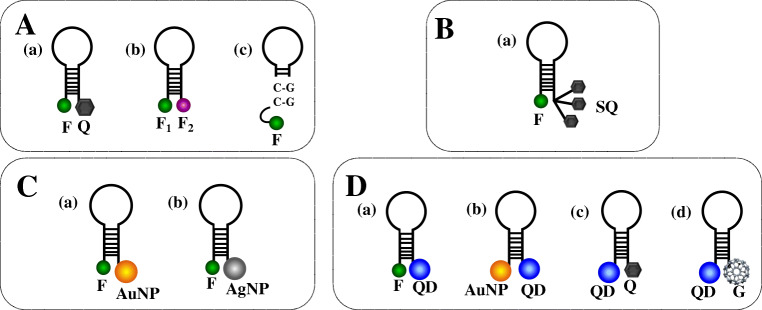
MB configurations. (A) Regular MBs: (a) MB modified with common signalling pairs (one fluorophore and one quencher), (b) MB with two different fluorophores, (c) MB with guanosine-rich sequences acting as the quencher. (B) Multiple-labelled MBs: (a) MB with a superquencher (SQ). (C) Nanomaterial-labelled MBs: (a) MB with a gold nanoparticle (AuNP) acting as the quencher; (b) MB with a silver nanoparticle (AgNP) acting as the quencher. (D) (a) MB with a quantum dot (QD) and a fluorophore acting as the energy donor and acceptor, respectively; (b) MB with a QD and an AuNP acting as the energy donor and acceptor, respectively; (c) MB with a QD and a quencher acting as the energy donor and acceptor, respectively; (d) MB with a QD and spherical fullerene (G) acting as the energy donor and acceptor, respectively

A typical signalling pair comprises a fluorophore and a quencher (Fig. 2A(a)), whose selection depends on the detector’s read-out system. MBs are instead functionalized with only two fluorophores (no quencher) (Fig. 2A(b)) to analyse intermolecular interactions among structured nucleic acids, including hairpin structures [46]. In this case, the MB/target hybridization significantly increases FRET between fluorophores, making dual-fluorophore-labelled MBs better suited for in vitro and in vivo sensing than regular MBs [47]. Guanosine residues in the stem portion of MB can replace the quencher by generating single-labelled MB systems (Fig. 2A(c)) [48]. Single-labelled MBs are cheaper and can be obtained with more straightforward functionalization procedures than dual-modified MBs and are particularly suited for biosensing applications involving solid supports. MB probes bearing multiple signalling labels in the same structure have been synthesized to enhance the fluorescence intensity. Yang et al. [49] reported an MB bearing a fluorophore and more quenchers by developing an SQ structure (Fig. 2B(a)), leading to a 320-fold increase of the fluorescent signal, far better than conventional MBs. Multiple-labelled MB probes may include one fluorophore at one end of the stem of the hairpin structure and two quenchers, such as guanine and organic quenchers, at the other end to obtain a double quenching MB [50]. The efficient transfer of energy from the fluorophore to quenchers in similar MBs can be exploited to detect DNA target 200–300 pM in concentration.

Some nanomaterials hold excellent quenching properties that significantly enhance the quenching efficiency compared to conventional quenchers used for MB probes (Fig. 2C). They are often used for nucleic acid target discrimination. Wang et al. [51] synthesized gold nanoparticle (AuNP)–based MBs (Fig. 2C(a)) exhibiting good stability and resistance to nuclease degradation and adequate quenching capacity and sensing performances for the detection of microRNA targets. Also, silver nanoparticles (AgNPs) exhibit similar quenching properties (Fig. 2C(b)) combined with an effective increase of the fluorescence generated when the fluorophore is separated from AgNPs as a consequence of MB conformational change [52]. MBs with AgNP quenchers combined with surface plasmon–coupled emission provided a 67-fold signal-to-background ratio and a discriminating capability for mismatch detection more than 25-fold compared to traditional surface plasmon–coupled emission.

Quantum dots (QDs) offer superior optical properties, such as broad absorption spectra and narrow emission spectra, high quantum yield, and remarkable photostability, which make them suitable for MB-based biosensing (Fig. 2D(a)) [53]. Depending on their size, QDs exhibit efficient fluorescence energy transfer that translates into better detection limits [54]. They are stable under high-salt conditions and critical pH values. MBs modified with both QDs and AuNPs (Fig. 2D(b)) overcome most of the limitations of conventional MBs that suffer from the modest half-life when used for in vivo applications. MBs modified with both QDs and AuNPs have been shown to promote DNA target sequences’ detection with a 1.4 fM limit of detection and the capacity to discriminate single-base mismatch and non-complementary sequences efficiently [50].

Carbon quantum dots (CQDs) offer low cytotoxicity, water solubility, and photostability. An MB with CQD and Black Hole Quencher 1 (BHQ1) connected to the stem ends (Fig. 2D(c)) has been used to detect microRNA-21 target [55]. The simple detection scheme adopted, exploiting the conventional conformational change of the MB probe triggered by the MB/target hybridization, provided a good sensitivity (300 pM detection limit) in detecting microRNA-21. The detection’s excellent specificity was proved by the perfect discrimination between microRNA-21 and single-mismatched sequence detection.

Graphene and graphene oxide (GO) represent emerging nanomaterials in biosensing. They combine exceptional optical, electrochemical, and electronic properties with an excellent fluorescence quenching activity [56–58]. Various MB-based biosensing approaches have been investigated by exploiting the spontaneous adsorption of DNA on GO via π–π stacking and hydrogen bonding [59]. Other carbon-based nanostructures, spherical fullerene (C_60_), also exhibit fluorescence quenching properties that have been used in combination with QDs to fabricate an MB nanosensor (Fig. 2D(d)) for DNA detection [60]. The quenching efficiency depends on the number and the size of C_60_ nanostructures next to each QD. Multiple QD-C_60_-labelled MB probes immobilized onto magnetic nanoparticle (MNP) amplify the fluorescence signal produced after target hybridization. The QD-C_60_-labelled MB-modified magnetic nanoparticles efficiently captured DNA targets in the sample then the magnetic force was applied to concentrate the MB/MNP complex to amplify the fluorescence signal for target quantification. The assay enabled rapid detection (<10 min) and, thanks to high signal-to-noise ratio produced by the QD-C_60_ pairs, a 100-fM detection limit in DNA detection.

MBs modified with a G-quadruplex scaffold (G4MB) combined with duplex-specific nuclease have been used to obtain a highly selective detection of microRNA-141 [61]. The duplex formed after the hybridization between the microRNA target and G4MB triggers the duplex’s enzymatic degradation. The duplex-specific nuclease cleaves only G4MB releasing the microRNA molecule from the duplex. The target microRNA is then recycled, causing the amplification of the fluorescence signal used to detect microRNA-141 with 1 pM detection limit. The G-quadruplex resistance to duplex-specific nuclease activity enables a reduction of false-positive signals leading to a low background signal.

## Origami-based structure design and mechanisms

**Fig. 3:**
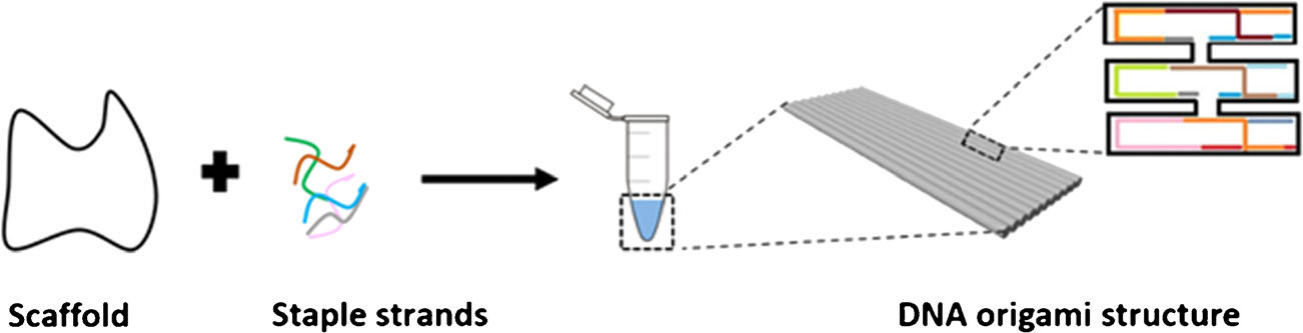
A pictorial description of DNA origami synthesis. It involves the annealing process among a long single-strand DNA (scaffold) and several hundreds of short ssDNA strands (staples) into 2D or 3D structures. From ref. [65]

Synthetic NAs have been used as engineering materials to create structures and functional devices with nanoscale precision [62]. Rothemund [63] demonstrated how folded DNA could be used as a versatile scaffold to assemble complex nanostructures with a bottom-up approach. DNA origami nanostructures provide new opportunities to develop innovative biosensing approaches (Fig. 3) [64]. A DNA origami is usually produced with a 7-kb ssDNA scaffold obtained from the circular genomic DNA of M13 bacteriophage (M13mp18). Hundreds of short ssDNAs (named staples) are then used to fold more extended scaffolds into a specific structure with extraordinary precision and high yield [13].

Atomic force microscopy (AFM), transmission electron microscopy (TEM), and dynamic light scattering (DLS) are used to confirm the optimal folding of components of the mixture [66].

Molecules and nanomaterials such as proteins [67], enzymes [68], and nanoparticles [69] can be incorporated into the origami structure, thus offering the opportunity to design new integrated functional interfaces. The extension of 2D scaffolds into 3D nanostructures [31] provides additional opportunities to fabricate engineered nanodevices with more complex structures and functions [70].

**Fig. 4:**
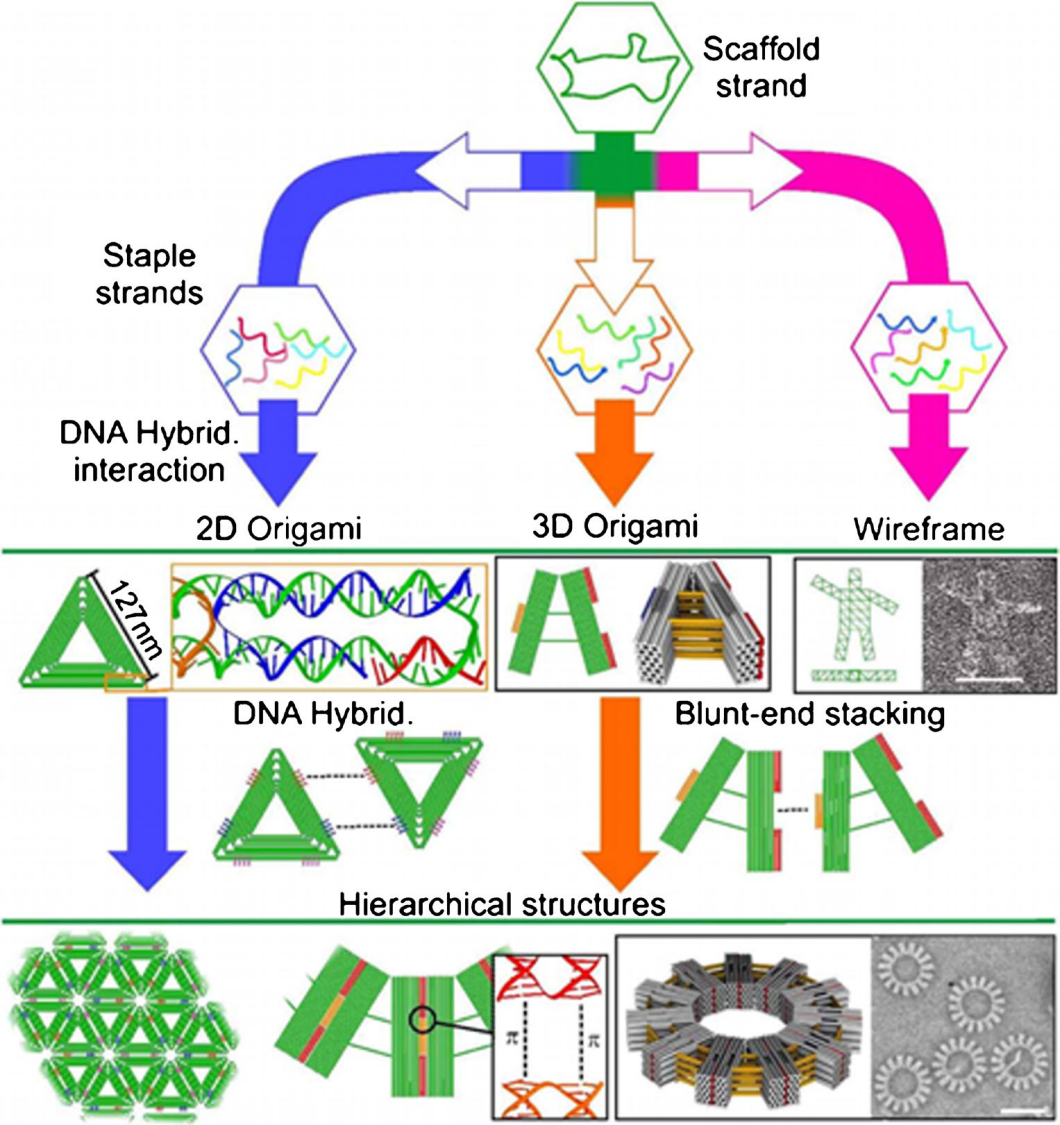
DNA origami assembly, from top to bottom: the scaffold strand is combined with different staple strands to build different 2D, 3D, or wireframe DNA origami structures. Such DNA origami can include sticky ends or blunt ends to assemble DNA origami units to form large-scale hierarchical 2D or 3D structures. Sticky ends are only shown for 2D DNA origami on the figure. From ref. [99]

The envisaged possibility of adopting biotechnology-based methods for the mass production of DNA origami components [71] can enable the broad applicability of DNA origami platforms for sensing [70]. The rational design of programmable DNA origami nanostructures with various levels of complexity (i.e., tetrahedral DNA, DNA nanopores, static and dynamic DNA origami, DNA nanopillars, DNA nanoantennas, DNA nanocubes, DNA wireframe, etc.) (Fig. 4) could contribute to overcoming labour-intensive and time-consuming approaches in biosensing, leading to the development of a new generation of specific, rapid, and high-throughput analytical platforms [72].

Tetrahedral DNAs are among the simplest 3D DNA nanostructures [73]. They can be used as cages to build more complicated nanostructures, exploiting functional moieties tailored to improve the biosensing capacity [74]. 3D shape, size, and double-stranded nature of the tetrahedron determine their optimal spacing and orientation on the sensing surface to favour target access and resistance to enzymatic degradation [75].

DNA origami nanostructures offer the opportunity to fabricate nanosensors that dynamically change their configuration in response to the analyte’s presence. The shuttling between an open and close conformation of the nanostructure can be triggered by modifying the reaction environment or the target detection [76]. The different configurations can also be associated with the generation of optical or electrochemical signals [77, 78]. DNA origami nanopores offer the opportunity to move sensing capacity at the single-molecule level [79]. Nanopores are typically produced with either solid-state methods or biological methods based on membrane-spanning protein’s reconstitution into a lipid bilayer. The nanopore geometry enables the selectivity of the detection method based on the physical size of the target molecule. The nanopore surface functionality instead allows for chemical specificity in nanopore-based detection. DNA origami nanopores have attracted considerable attention due to their peculiar features, allowing tuning the synthesized nanopores’ size and chemically modifying their surface. Recent literature has paid great attention to possibilities offered by DNA origami nanopores. Here, we emphasize the recent progress and innovation in the context of DNA origami nanopores compared with other DNA origami nanostructures.

Small solid-state nanopores are not easily obtained while biological nanopores are produced with atomic control, but their use for the detection of large molecules including double-stranded DNA and protein is still limited. DNA origami nanopores open new perspectives for the production of nanopores with accurate geometry and chemical functionality [80]. Two different approaches have been adopted to combine DNA origami features with nanopore technology [81]. DNA origami nanostructures have been trapped at the mouth of solid-state nanopores, thereby fabricating hybrid DNA origami-solid state nanopores [82]. DNA origami structures are, instead, inserted into a lipid bilayer membrane either by using hydrophobic moieties or streptavidin binding to biotinylated nanopores [83]. The latter approach leads to structures that more closely mimic membrane proteins’ behaviour controlling the transport of molecules and ions through cell membranes.

The ability to modulate hybrid DNA origami nanopores’ size allows extending the potential of nanopore technologies into biosensing and point-of-care diagnostics. The translocation of a single molecule through the nanopore modulates the electric or electro-optical detected signal, enabling selective detection and quantification [84]. Programmability and biocompatibility make DNA origami nanostructures excellent molecular systems for in vivo biomedical applications [85]. These include the biosensing of biomarkers in clinically relevant fluids coupled with a nanopore read-out [84]. Such a biosensing approach has been applied to detect C-reactive protein (CRP) in human plasma using a concentric square DNA origami bearing a target-specific aptamer within its central cavity. The aptamer-functionalized DNA origami captures CRP, and the detected electric signal allows distinguishing between the occupied and unoccupied DNA origami’s translocation fingerprints.

Hybrid DNA origami nanopores display peculiar features for single-molecule detection [86] since they allow to precisely modulate the size of the nanopore to establish size-dependent discrimination of molecules or to enable the translocation of molecules with a selected arrangement (e.g., folded DNA vs unfolded DNA) [87]. However, constraints related to the length of the origami scaffold and the adequate number of membrane-anchoring positions are still present in DNA nanopores’ production [83]. The widest synthetic transmembrane DNA origami nanopore so far obtained (inner diameter 9.6 nm; outer diameter 22 nm; length 32 nm) is based on a double-layered hexagonal DNA origami structure [88]. Such nanopores allow the translocation of large proteins (more than 150 kDa). They also allow the management of a programmable functionality of the whole system to drive the nanopore’s opening thus exposing lipid moieties that permit membrane insertion.

Densely packed 3D DNA nanostructures have also been considered for their stability and resistance to nuclease and high temperatures [89–91]. Seeman [92] reported the pioneering example of a similar structure consisting of a discrete 3D DNA structure forming a nanocube. Later on, Shih and co-workers [93] synthesized a nanoscale octahedron using a ssDNA. Both systems were 3D structures whose dynamic properties were triggered by the components used to form the structure. Other triggers such as temperature [94], pH change [95], and light [96] have also been investigated to activate DNA origami responses with different shapes.

DNA nanostructures have also been employed for in vivo imaging. Bhatia and co-workers [97] synthesized a DNA icosahedron encapsulating a fluorescent biopolymer to monitor pH in *Caenorhabditis elegans* (*C. elegans*), whereas Kim and co-workers [98] used a fluorescently labelled DNA tetrahedron for imaging sentinel lymph nodes in mouse models.

DNA nanostructures can improve the antifouling properties of biosensing surfaces [100]. The minimization of the non-specific adsorption of biological fluids’ components [101] on the surface of a biosensor improves sensitivity and selectivity of biosensing assays, thus allowing the detection of ultra-low-concentrated biomarkers in complex biofluids [102, 103]. The ability to detect biomarkers directly in the biological fluids is a pre-requisite to developing challenging biosensing applications for clinical diagnostics [104–106]. With this perspective, surface plasmon resonance (SPR) has been used to detect human thrombin with a 3D DNA origami structure modified with an aptamer [107]. The DNA origami allows arranging the thrombin-specific aptamer on the SPR sensor’s surface with nanoscale precision and contributes to favouring the thrombin detection with a broad linear detection range.

## Molecular beacons and DNA origami in biosensing: a comparison and peculiar applications

MB and origami scaffolds are here compared to highlight the pros and cons in biosensing of structures resulting from the programmable design of interactions between DNA sequences. In this respect, MBs represent the simplest structure discussed, whereas multidimensional origamis are the most complex systems whose structure and function can be designed mostly based on Watson-Crick base pair.

The application of the conventional stem-loop hairpin structure of MB probes, including fluorophore/quencher signalling pair, allows for measuring an instantaneous signal emission directly in solution, without removing unreacted MB molecules, in one single “mix-and-measure” step. MB-based sensing approaches can operate in the open-to signal or close-to signal configurations, depending on the design and the conformation of the MB probe [43]. The open-to signal configuration enables detecting the fluorescent signal related to the target molecule after linearising the stem-loop hairpin MB structure, thus defining turn-on biosensors. Instead, the fluorescence emission is initially maximum in the close-to signal state due to the MB interaction with a helper oligonucleotide sequence. The interaction between the target and helper sequences triggers the hairpin probe’s closure, thus turning off the fluorescent signal. Such a mechanism identifies turn-off biosensors that operate with lower detection sensitivity than turn-on biosensors [108]. A turn-off biosensor may report a further loss of fluorescence due to the unspecific interaction of molecules available in the biological sample with the MB probe. A similar interaction causes the unspecific quenching of the fluorescence signal. Conversely, a turn-on biosensor generates an intense signal when a specific interaction between target and MB probe is established.

Besides MB-based homogeneous biosensing methods, stem-loop hairpin structures can also operate as sensing probes after their immobilization on surfaces [108]. In this case, optical or electrochemical signalling tags are introduced to label one of the stems of the hairpin-shaped MB, while the other stem is anchored on a solid substrate. A specific signal is produced due to the structure’s conformational change when the hybridization between the target and the loop region occurs. Similar MB-based biosensors have been largely applied to detect DNAs [109, 110], microRNAs [111], proteins [112], metal ions [113–115], and pathogens/viruses [116].

MB-based homogeneous biosensing is faster than solid-state biosensing because hybridization reactions are kinetically favoured. MB-based solid-state biosensors’ analytical performances are also negatively affected by steric hindrance and slow kinetics of the diffusion of target molecules to the sensing surface. Moreover, the fabrication of MB-based solid-state biosensors involves labourious protocols for the immobilization of the MB probe, ensuring an optimal spatial orientation and availability of the anchored probe [117]. Nevertheless, MB-based solid-state biosensing platforms provide optimal signal-to-background ratio, resulting in a sensitivity enhancement. They can also be regenerated with no significant attenuation of the signal after the regeneration. MB-based solid-state biosensing can take on the photobleaching phenomenon of dye-labelled probes [118].

DNA origami scaffolds help overcome the limitations of MB-based solid-state biosensors related to the constrained availability of the sensing probe to the target. Such a possibility improves the performance of the biosensing device, particularly when its surface is exposed to complex biological fluids, and optimal control of probe packing density and target hybridization efficiency is required [119]. The sensitivity of electrochemical biosensors in NA detection is greatly influenced by the limited accessibility of the probe immobilized on the electrode surface due to surface crowding and charge effects. Such biosensors greatly benefit from replacing MB probes with rigid DNA tetrahedra that reduce steric hindrance and promote suitable probe spacing and orientation [120].

Liu et al. [121] fabricated a 3D DNA tetrahedron using a multistranded approach. In this case, the scaffold strand of conventional origami structures is not used and a stem-loop probe and thiol moieties are introduced to modify the top corner and the bottom of the 3D DNA tetrahedron, respectively. A ferrocene tag modified the stem-loop probe to allow electrochemical biosensing. The hybridization of microRNA-21 (lung cancer biomarker) with the stem-loop structure enabled a conformational change of the probe giving rise to a detectable differential pulse voltammetry output caused by the interaction of the ferrocene tag with the gold electrode. The new detection approach allowed detecting microRNA-21 with 10 pM LOD and linear range response spanning from 100 pM to 1 μM.

The use of conventional MB scaffolds for the real-time monitoring of targets in living cells represents another challenging application hindered by limitations in cell membrane permeabilization and efficient delivery of the MB probe into the cytoplasm [122]. Several strategies have been proposed to make MB structures more readily applicable for in vivo diagnosis [122]. Tay et al. [123] proposed an innovative combination of a 3D DNA pyramid-shaped nanoshell and an MB probe bearing a fluorophore/quencher signalling pair. The complex was internalized in the cell and protected from non-discriminatory enzymatic digestion. A similar approach was also used to develop a nanoprobe to detect cancer-related messenger RNA in living cells [124]. The experiments mentioned above demonstrate that DNA origami structures combined with stem-loop MB sequences provide advanced diagnostic performances because their cellular permeabilization in vivo is favoured compared to conventional MBs and because they prevent nuclease degradation of MBs and interaction with DNA-binding proteins leading to false-positive signals.

As already mentioned, many attempts have been made to extend the modest half-life of conventional MBs for in vivo applications by using nanomaterial-based signalling and quenching moieties. The modification of the MB backbone or the use of DNA mimics [125] (e.g., PNA or LNA) [26, 126] further contributes to overcoming limitations of traditional MBs for in vivo biosensing. The stability of PNA-DNA duplexes has been also proven to favour the incorporation of PNA into a rectangular DNA origami [127].

DNA origami nanostructures offer additional possibilities for biosensing compared with MBs thanks to the capacity to regulate the activity of enzymes encapsulated in the nanostructure [128]. A DNA origami nanovault allows loading the enzyme in the DNA nanostructure cavity and controlling the access to the substrate molecules [68]. However, it is to underline that the use of such nanostructures in in vitro culture environments may be critical and conditions that prevent the loss of nanostructure integrity should be accurately selected and managed [129].

Conventional MB probes operate based on a one-to-one signal read-out approach (i.e., one target molecule recognizes and hybridizes with one MB probe, thus triggering an output signal) [130]. MBs’ ability to accurately monitor low-abundant levels is limited due to the poor sensitivity and the relatively low signal-to-noise ratio. Such a peculiar feature is particularly critical in designing advanced and high-throughput MB-based biosensing platforms for microRNA detection and quantification [131]. The ability to monitor the microRNA expression profile is fundamental for clinical diagnostic purposes. Therefore, efforts have been paid in identifying solutions for optimal use of MB biosensing features in microRNA detection. In this context, Wang et al. [132] reported a relevant application of an electrochemiluminescence biosensor using a DNA hairpin probe and graphene oxide to detect a femtomolar microRNA target (miR-21) in human adenocarcinoma cells (A549).

The multiplexed detection of exosomal microRNAs can be used to develop innovative diagnostic approaches based on the analysis of liquid biopsies [104]. In this context, MBs have been employed for the simultaneous detection of miR-21, miR-375, and miR-27a in exosomes from breast cancer cells [133]. Although multiplexed detection and cell permeability are critical issues when dealing with conventional MBs, MB probes have been shown to penetrate exosomes and hybridize microRNA targets inside the exosomal environment. The detection is performed with nanomolar sensitivity to be compared with the sub-picomolar sensitivity reported for origami-based structures in similar applications. Also, the immunodetection of exosomal proteins has been demonstrated using a similar MB-based approach [134].

The optical detection of microRNA has also been performed with the DNA “points accumulation for imaging in nanoscale topography” (PAINT) approach using synthetic DNA nanostructures as a programmable microRNA capture array [135–137]. The multiplexed PAINT imaging approach quantified endogenous microRNAs in total RNA extract from HeLa cells and achieved the multiplexed detection of microRNAs in either 30 pM or 100 pM concentrations.

The plasmonic enhancement in the gap between metal nanoparticles or collective chiral plasmonic systems can be exploited to improve the optical detection process’s sensitivity significantly. DNA origami nanostructures help precisely tune the interparticle gap between assembled plasmonic nanoparticles by exhibiting drastic SERS effects allowing single-molecule detection [138]. Funck and co-workers [139] developed a chiral optical nanodevice consisting of two arms each carrying one gold nanorod. The two arms were connected by ssDNAs allowing the turning of the two arms. The resulting structure consisted of a cross-shaped DNA origami whose structure’s conformation changes introduced modifications in the detected plasmonic circular dichroism spectrum. In the absence of the target, the two levers were linked by a single-stranded hinge and included complementary single-stranded extensions on each lever initially prevented by the hybridization of a blocking oligonucleotide. The hybridization of the picomolar viral RNA displaced the oligonucleotides and established the closed configuration of the nanodevice.

Metal nanoparticles can constitute a plasmonic hotspot in the DNA scaffold [70, 140] to favour enhancing the detected signal. DNA origami nanoantennas have been used to locate AuNP pairs at a gap of 12–17 nm, thus obtaining a 5000-fold fluorescence enhancement and single-molecule detection [141]. A single-molecule platform for the label-free detection of DNA and RNA Zika virus sequences by confocal microscopy has been fabricated [142], combining DNA origami with an MB structure and Au/Si nanoparticles. The optical nanoantenna produced a plasmonic hotspot on a DNA origami scaffold, on the bottom of which the dye-quencher pairs of an MB induced the reduction of fluorescence emission. The platform detected the target sequences in human serum with 1 nM sensitivity and enabled multiplexed detection by combining multiple antenna designs.

Table 1 summarizes the most relevant MBs and DNA origami systems used in biosensing.

**Table 1:** Examples of different MBs and DNA origami for biosensing applications

	Design	Features	Target	Ref.
Molecular beacons (MBs)	Dual-fluorophore-labelled MBs	Dual FRET signal In vitro and in vivo sensing Signal-to-background ratio enhancement	Non-repetitive regions of MUC4 gene	[47]
	Single-labelled MBs	Low-cost synthesis Simple functionalization Rapid and multiplexed detection	Tumour suppressor genes (p16, p53)	[48]
	Multiple-labelled MBs (SQs)	Synergistic quenching effect High sensitivity and accuracy Rapid analysis Low-cost detection	HBV and HIV sequences in serum	[50]
	Nanomaterials (AuNPs/AgNPs)-MBs	Resistance to nuclease cleavage High quenching efficiency High sensitivity and specificity High specificity with mismatches and homologous discrimination	microRNAs (miR-21-5p, miR-92a-3p) Sequences of *Staphylococcus aureus* femA methicillin resistance genes in serum	[51] [52]
	Quantum dots (QD-AuNPs/CQD)-MBs	FRET efficiency Low detection limit Low cytotoxicity Water solubility Photostability	Full complementary, single nucleotide and non-complementary DNAs miR-21	[143] [55]
	Graphene (fullerene C60/GO)-MBs	High quenching efficiency Rapid, robust sensing	DNA miR-21	[60] [132]
	G-quadruplex (G4)-MBs	Resistance to nuclease cleavage False-positive reduction Low background signal Single-base selectivity Multiplexed detection	microRNAs (miR-141, miR-429, miR-200b, miR-21)	[61]
DNA origami	Nanopore on concentric square structures	Single-molecule detection Multimodal read-out signal	hCRP in plasma	[84]
	3D tetrahedron	Low detection limit Cost-effective read-out signal	miR-21	[121]
	3D pyramid-shaped nanoshell-MB	Live cell imaging Resistance to nuclease cleavage	mRNA	[123]
	Tetrahedron-MBs	Live cell imaging	TK1 mRNA	[124]
	Nanocage	Catalytic activity improvement No enzyme aggregation Cost-effective signal transducer	HRP, MDH, G6PDH, LDH, GOx, β-Gal	[128]
	Nano-arrays	Nanometre-precise spacing Multiplexed detection Single-molecule detection Super-resolution imaging	microRNAs (miR-342-3p, miR-21-5p, miR-16-5p, miR-145-5p, miR-375, miR-24-3p, miR-378a-3p, miR-221-3p, miR-186-5p, miR-155-5p, miR-642b-3p, let-7a-5p, miR-485-3p, miR-372-3p, miR-491-5p, miR-154-5p)	[135]
	Cross-shape-AuNRs	Low detection limit Large signal variation with conformational change	Viral RNA in serum	[139]
	Nanoantenna-AuNPs	Quantum-yield improvement Reduced interparticle distance Single-molecule detection	DNA	[141]
	Nanoantenna-MBs-Au/Si NPs	No signal amplification required Plasmonic hotspot for sensing enhancement Single-molecule detection	Zika virus DNA and RNA in serum	[142]

*MUC4*, Mucin 4 gene; *hCRP*, human C-reactive protein; *mRNA*, messenger RNA; *TK1*, Thymidine kinase 1; *HRP*, horseradish peroxidase; *MDH*, malic dehydrogenase; *G6PDH*, glucose-6-phosphate dehydrogenase; *LDH*, lactic dehydrogenase; *GOx*, glucose oxidase; *β-Gal*, β-galactosidase

## Conclusions and perspectives

Over the past two decades, NA sequences have been used to assemble a plethora of nanostructures exploiting complementary base pairing, straightforward programmability of interactions between NA sequences, and NA structure rigidity and stability. The efforts paid have identified NA nanotechnology as an emerging and fascinating new field with substantial implications for biosensing. Here, we have summarized recent advances in MBs and DNA origami structures by critically evaluating their intrinsic role as biosensing elements. MBs belong to a more conventional class of NA structures used in biosensing, whereas DNA origami structures are fabricated by fully exploiting possibilities offered by NA nanotechnology.

MB-based structures detect NAs (including DNA and microRNA) with satisfactory performances. Different strategies have been adopted to fabricate MB devices well-performing in biosensing. These include MB self-assembly or combination with nanomaterials such as AuNPs, QDs, and superquenchers.

Pushing the limit of detection of MB platforms to the sub-picomolar range would bring more opportunities to detect targets such as circulating tumour DNA (ctDNAs), viral DNA or RNAs, and small molecules available in biological fluids at low concentrations. Along this way, conventional hairpin structures, acting as strand-mediated signal amplifiers, combined with 3D DNA origami structures, enhance the signal amplification, thus allowing achieving exceptional detection limits through continuous DNA amplification reactions.

The challenging label-free single-molecule detection of complex targets, such as viruses or exosomes, in biological fluids (plasma or serum) remain a prerogative of the more recent origami nanostructures. Moreover, these structures’ programmable nature at the nanoscale level enables multiplexed detection of more than one biomarker in a single assay.

Origami nanostructures help improve the control of probe spacing and orientation at the biosensing interface, improving detection sensitivity and selectivity. The stability in vivo of origami nanostructures is better compared with MBs. Besides, they exhibit unpredictable properties such as permeability across the cellular membranes, leading to several exciting applications when combined with other nanomaterials.

Despite all these examples, some key challenges still need to be addressed before routinely translating DNA origami nanostructures into robust devices for biosensing applications. The folding of DNA origami nanostructures requires thermal annealing in the presence of a minimum concentration of cations to prevail the negative charge-repulsion of the backbone. Origami nanostructures are usually formed in a buffer comprising Mg^2+^ at a concentration of about one order of magnitude higher than its concentration in blood and tissues. Such a difference may cause the denaturation of the nanostructure in biological environments even though various DNA origami nanostructures keeping their integrity in low magnesium buffers have been described [144]. Such a requirement still limits their use for in vivo diagnosis even though simpler structures operating as nucleic acid nanoswitch have been used to rapidly measure immunoglobulin (IgG and IgE) levels directly in blood serum and other bodily fluids [145].

DNA nanostructures are assembled from a scaffold structure held in place by many ssDNA staples. The large number of ssDNA sequences required to assemble such structures hampers the massive production of origami-based biosensors due to production and cost restrictions. However, new possibilities paving the way toward the large-scale production of DNA nanostructures have been recently identified. Praetorius et al. defined an innovative protocol for litre-scale ssDNA production and large-scale DNA origami assembly [71]. The protocol is compatible with existing DNA origami design frameworks and uses bacteriophages to generate ssDNA precursors. Precursors contains target strand sequences interleaved with self-excising DNAzyme cassettes. A compromise between design simplicity and complexity of the explicated function obtained with origami structures assembled using a low number of DNA strands designed with user-friendly design platforms and produced with automated synthesis will be a breakthrough to make such scaffolds more accessible for biosensing applications. Despite the above still unresolved challenges, it is surprising to see how many multidisciplinary approaches among different fields have converged to deal with these issues continually proposing new strategies and ideas to extend DNA molecular assembly, detection, and applications.

MBs and DNA origami nanostructures, each with own strengths and weaknesses (Table 2), will help understand complex biological systems contributing to reveal nanoscale-level molecular interactions while providing a route to smarter, more accurate, more sensitive NA devices able to solve real-life problems in human healthcare.

**Table 2:** Advantages and limitations of MBs and DNA origami-based structures in biosensing

DNA-based structures	Advantages	Limitations
Molecular beacons (MBs)	Higher selectivity than linear DNA probes Photo-stability owing to quencher and dye labels Intrinsic sensing mechanism Good biorecognition Chemical simplicity Small size Good thermal stability Cost-effective	High signal background due to incomplete quenching Limited sensitivity Low efficiency Labelling requirement No long-term in vivo applications Toxicity of labels Reduced availability of the sensing probe to target at the solid-liquid interface One-to-one signal read-out
DNA origami	Single-step folding process in high yields Specific site addressability Easy modification Wide applicability Enhancement in sensitivity with single-molecule detection Resistance to fouling in complex matrices Improved accessibility of sensing probe to target at the solid-liquid interface Significant resistance to nuclease degradation Multiple hybridization ratio with target molecules	Conventional and expensive characterization techniques Salt concentration in the biological environment may alter the origami stability Limited size depending on the scaffold length High ion permeability

## References

[1] Kogikoski S, Paschoalino WJ, Kubota LT (2018). Supramolecular DNA origami nanostructures for use in bioanalytical applications. TrAC - Trends Anal Chem.

[2] Chen Y-J, Groves B, Muscat RA, Seelig G (2015). DNA nanotechnology from the test tube to the cell. Nat Nanotechnol.

[3] Du Y, Dong S (2017). Nucleic acid biosensors: recent advances and perspectives. Anal Chem.

[4] Rossetti M, Porchetta A (2018). Allosterically regulated DNA-based switches: from design to bioanalytical applications. Anal Chim Acta.

[5] Zhang P, Jiang J, Yuan R, Zhuo Y, Chai Y (2018). Highly ordered and field-free 3D DNA nanostructure: the next generation of DNA Nanomachine for rapid single-step sensing. J Am Chem Soc.

[6] Li J, Green AA, Yan H, Fan C (2017). Engineering nucleic acid structures for programmable molecular circuitry and intracellular biocomputation. Nat Chem.

[7] Genot AJ, Baccouche A, Sieskind R, Aubert-Kato N, Bredeche N, Bartolo JF, Taly V, Fujii T, Rondelez Y (2016). High-resolution mapping of bifurcations in nonlinear biochemical circuits. Nat Chem.

[8] Kopperger E, List J, Madhira S, Rothfischer F, Lamb DC, Simmel FC (2018). A self-assembled nanoscale robotic arm controlled by electric fields. Science.

[9] Jung C, Allen PB, Ellington AD (2016). A stochastic DNA walker that traverses a microparticle surface. Nat Nanotechnol.

[10] Yue L, Wang S, Willner I (2019). Three-dimensional nucleic-acid-based constitutional dynamic networks: enhancing diversity through complexity of the systems. J Am Chem Soc.

[11] Zhou Z, Yue L, Wang S, Lehn J-M, Willner I (2018). DNA-based multiconstituent dynamic networks: hierarchical adaptive control over the composition and cooperative catalytic functions of the systems. J Am Chem Soc.

[12] García-Fernández A, Megens RP, Villarino L, Roelfes G (2016). DNA-accelerated copper catalysis of Friedel–Crafts conjugate addition/enantioselective protonation reactions in water. J Am Chem Soc.

[13] Seeman NC, Sleiman HF (2017). DNA nanotechnology. Nat Rev Mater.

[14] Hong F, Zhang F, Liu Y, Yan H (2017). DNA origami: scaffolds for creating higher order structures. Chem Rev.

[15] Chidchob P, Sleiman HF (2018). Recent advances in DNA nanotechnology. Curr Opin Chem Biol.

[16] Teles FRR, Fonseca LP (2008). Trends in DNA biosensors. Talanta.

[17] Labuda J, Brett AMO, Evtugyn G, Fojta M, Mascini M, Ozsoz M, et al. Electrochemical nucleic acid-based biosensors: concepts, terms, and methodology (IUPAC Technical Report). Pure Appl Chem. 82:1161–87. 10.1351/PAC-REP-09-08-16.

[18] Yola ML, Eren T, Atar N (2014). A novel and sensitive electrochemical DNA biosensor based on Fe@Au nanoparticles decorated graphene oxide. Electrochim Acta.

[19] Zhang Y, Huang L (2012). Label-free electrochemical DNA biosensor based on a glassy carbon electrode modified with gold nanoparticles, polythionine, and graphene. Microchim Acta.

[20] Wang M, Wiraja C, Wee M, Yeo D, Hu L, Xu C (2018). Hairpin-structured probe conjugated nano-graphene oxide for the cellular detection of connective tissue growth factor mRNA. Anal Chim Acta.

[21] Meng HM, Liu H, Kuai H, Peng R, Mo L, Zhang XB (2016). Aptamer-integrated DNA nanostructures for biosensing, bioimaging and cancer therapy. Chem Soc Rev.

[22] Dunn MR, Jimenez RM, Chaput JC (2017). Analysis of aptamer discovery and technology. Nat Rev Chem.

[23] Nguyen V-T, Kwon YS, Gu MB (2017). Aptamer-based environmental biosensors for small molecule contaminants. Curr Opin Biotechnol.

[24] Poolsup S, Kim C-Y (2017). Therapeutic applications of synthetic nucleic acid aptamers. Curr Opin Biotechnol.

[25] Briones C, Moreno M (2012). Applications of peptide nucleic acids (PNAs) and locked nucleic acids (LNAs) in biosensor development. Anal Bioanal Chem.

[26] D’Agata R, Giuffrida MC, Spoto G. Peptide nucleic acid-based biosensors for cancer diagnosis. Molecules. 2017:22. 10.3390/molecules22111951. 10.3390/molecules22111951 PMC6150339 29137122

[27] Li J, Zhou J, Liu T, Chen S, Li J, Yang H (2018). Circular DNA: a stable probe for highly efficient mRNA imaging and gene therapy in living cells. Chem Commun.

[28] Ge Z, Lin M, Wang P, Pei H, Yan J, Shi J, Huang Q, He D, Fan C, Zuo X (2014). Hybridization chain reaction amplification of microRNA detection with a tetrahedral DNA nanostructure-based electrochemical biosensor. Anal Chem.

[29] Borum RM, Jokerst JV (2021). Hybridizing clinical translatability with enzyme-free DNA signal amplifiers: recent advances in nucleic acid detection and imaging. Biomater Sci.

[30] Santangelo PJ, Nix B, Tsourkas A, Bao G. Dual FRET molecular beacons for mRNA detection in living cells. Nucleic Acids Res. 2004;32:e57–7. 10.1093/nar/gnh062. 10.1093/nar/gnh062 PMC390379 15084672

[31] Douglas SM, Dietz H, Liedl T, Högberg B, Graf F, Shih WM (2009). Self-assembly of DNA into nanoscale three-dimensional shapes. Nature.

[32] Hu Q, Li H, Wang L, Gu H, Fan C (2019). DNA nanotechnology-enabled drug delivery systems. Chem Rev.

[33] Pei H, Liang L, Yao G, Li J, Huang Q, Fan C (2012). Reconfigurable three-dimensional DNA nanostructures for the construction of intracellular logic sensors. Angew Chem Int Ed.

[34] Shaw A, Benson E, Högberg B (2015). Purification of functionalized DNA origami nanostructures. ACS Nano.

[35] Wagenbauer KF, Engelhardt FAS, Stahl E, Hechtl VK, Stömmer P, Seebacher F, Meregalli L, Ketterer P, Gerling T, Dietz H (2017). How we make DNA origami. Chembiochem.

[36] Ranallo S, Porchetta A, Ricci F (2019). DNA-based scaffolds for sensing applications. Anal Chem.

[37] Tyagi S, Kramer FR (1996). Molecular beacons: probes that fluoresce upon hybridization. Nat Biotechnol.

[38] Sokol DL, Zhang X, Lu P, Gewirtz AM (1998). Real time detection of DNA.RNA hybridization in living cells. Proc Natl Acad Sci U S A.

[39] Vet JA, Majithia AR, Marras SA, Tyagi S, Dube S, Poiesz BJ, Kramer FR (1999). Multiplex detection of four pathogenic retroviruses using molecular beacons. Proc Natl Acad Sci U S A.

[40] Jordens JZ, Lanham S, Pickett MA, Amarasekara S, Abeywickrema I, Watt PJ (2000). Amplification with molecular beacon primers and reverse line blotting for the detection and typing of human papillomaviruses. J Virol Methods.

[41] Giuffrida MC, Zanoli LM, D’Agata R, Finotti A, Gambari R, Spoto G (2015). Isothermal circular-strand-displacement polymerization of DNA and microRNA in digital microfluidic devices. Anal Bioanal Chem.

[42] Vet JAM, Marras SAE (2005). Design and optimization of molecular beacon real-time polymerase chain reaction assays. Methods Mol Biol.

[43] Bidar N, Amini M, Oroojalian F, Baradaran B, Hosseini SS, Shahbazi M-A, Hashemzaei M, Mokhtarzadeh A, Hamblin MR, de la Guardia M (2021). Molecular beacon strategies for sensing purpose. TrAC Trends Anal Chem.

[44] Huang K, Martí AA (2012). Recent trends in molecular beacon design and applications. Anal Bioanal Chem.

[45] Zheng J, Yang R, Shi M, Wu C, Fang X, Li Y, Li J, Tan W (2015). Rationally designed molecular beacons for bioanalytical and biomedical applications. Chem Soc Rev.

[46] Yang CJ, Tan W (2013). Molecular beacons.

[47] Mao S, Ying Y, Wu X, Krueger CJ, Chen AK. CRISPR/dual-FRET molecular beacon for sensitive live-cell imaging of non-repetitive genomic loci. Nucleic Acids Res. 2019;47:e131–1. 10.1093/nar/gkz752. 10.1093/nar/gkz752 PMC6847002 31504824

[48] Xiang D-S, Zhai K, Wang L-Z (2013). Multiplexed DNA detection with a composite molecular beacon based on guanine-quenching. Analyst.

[49] Yang CJ, Lin H, Tan W (2005). Molecular assembly of superquenchers in signaling molecular interactions. J Am Chem Soc.

[50] Xiang D, Li F, Wu C, Shi B, Zhai K (2017). The G-BHQ synergistic effect: improved double quenching molecular beacons based on guanine and Black Hole Quencher for sensitive simultaneous detection of two DNAs. Talanta.

[51] Wang B, You Z, Ren D (2019). Target-assisted FRET signal amplification for ultrasensitive detection of microRNA. Analyst.

[52] Chen J, Luo Z, Wang Y, Huang Z, Li Y, Duan Y (2018). DNA specificity detection with high discrimination performance in silver nanoparticle coupled directional fluorescence spectrometry. Sensors Actuators B Chem.

[53] Liu H, Shu W, Liu Z, Zhang B, Feng H, Chen Y (2017). A simple method of constructing microfluidic solid-state quantum dot molecular beacon array for label-free DNA detection. Microfluid Nanofluidics.

[54] Adegoke O, Park EY (2016). The use of nanocrystal quantum dot as fluorophore reporters in molecular beacon-based assays. Nano Converg.

[55] Mahani M, Mousapour Z, Divsar F, Nomani A, Ju H (2019). A carbon dot and molecular beacon based fluorometric sensor for the cancer marker microRNA-21. Microchim Acta.

[56] Kasry A, Ardakani AA, Tulevski GS, Menges B, Copel M, Vyklicky L (2012). Highly efficient fluorescence quenching with graphene. J Phys Chem C.

[57] Wang N, Liu ZX, Li RS, Zhang HZ, Huang CZ, Wang J (2017). The aggregation induced emission quenching of graphene quantum dots for visualizing the dynamic invasions of cobalt(ii) into living cells. J Mater Chem B.

[58] Liu X, Wu Y, Wu X, Zhao JX (2019). A graphene oxide-based fluorescence assay for the sensitive detection of DNA exonuclease enzymatic activity. Analyst.

[59] Liu B, Salgado S, Maheshwari V, Liu J (2016). DNA adsorbed on graphene and graphene oxide: fundamental interactions, desorption and applications. Curr Opin Colloid Interface Sci.

[60] Liu Y, Kannegulla A, Wu B, Cheng L-J (2018). Quantum dot fullerene-based molecular beacon nanosensors for rapid, highly sensitive nucleic acid detection. ACS Appl Mater Interfaces.

[61] Zhou H, Yang C, Chen H, Li X, Li Y, Fan X (2017). A simple G-quadruplex molecular beacon-based biosensor for highly selective detection of microRNA. Biosens Bioelectron.

[62] Seeman NC (2003). DNA in a material world. Nature.

[63] Rothemund PWK (2006). Folding DNA to create nanoscale shapes and patterns. Nature.

[64] Wang P, Meyer TA, Pan V, Dutta PK, Ke Y (2017). The beauty and utility of DNA origami. Chem.

[65] Loretan M, Domljanovic I, Lakatos M, Rüegg C, Acuna GP (2020). DNA origami as emerging technology for the engineering of fluorescent and plasmonic-based biosensors. Materials (Basel).

[66] Castro CE, Kilchherr F, Kim D-N, Shiao EL, Wauer T, Wortmann P, Bathe M, Dietz H (2011). A primer to scaffolded DNA origami. Nat Methods.

[67] Saccà B, Meyer R, Erkelenz M, Kiko K, Arndt A, Schroeder H, Rabe KS, Niemeyer CM (2010). Orthogonal protein decoration of DNA origami. Angew Chem Int Ed Engl.

[68] Grossi G, Dalgaard Ebbesen Jepsen M, Kjems J, Andersen ES (2017). Control of enzyme reactions by a reconfigurable DNA nanovault. Nat Commun.

[69] Thacker VV, Herrmann LO, Sigle DO, Zhang T, Liedl T, Baumberg JJ, Keyser UF (2014). DNA origami based assembly of gold nanoparticle dimers for surface-enhanced Raman scattering. Nat Commun.

[70] Loescher S, Groeer S, Walther A (2018). 3D DNA origami nanoparticles: from basic design principles to emerging applications in soft matter and (bio-)nanosciences. Angew Chem Int Ed Engl.

[71] Praetorius F, Kick B, Behler KL, Honemann MN, Weuster-Botz D, Dietz H (2017). Biotechnological mass production of DNA origami. Nature.

[72] Rutten I, Daems D, Lammertyn J (2020). Boosting biomolecular interactions through DNA origami nano-tailored biosensing interfaces. J Mater Chem B.

[73] Goodman RP, Schaap IAT, Tardin CF, Erben CM, Berry RM, Schmidt CF, Turberfield AJ (2005). Rapid chiral assembly of rigid DNA building blocks for molecular nanofabrication. Science.

[74] Goodman RP, Berry RM, Turberfield AJ. The single-step synthesis of a DNA tetrahedron. Chem Commun. 2004:1372–3. 10.1039/B402293A. 10.1039/b402293a 15179470

[75] Pei H, Lu N, Wen Y, Song S, Liu Y, Yan H, Fan C (2010). A DNA nanostructure-based biomolecular probe carrier platform for electrochemical biosensing. Adv Mater.

[76] Ijäs H, Nummelin S, Shen B, Kostiainen M, Linko V (2018). Dynamic DNA origami devices: from strand-displacement reactions to external-stimuli responsive systems. Int J Mol Sci.

[77] Goetzfried MA, Vogele K, Mückl A, Kaiser M, Holland NB, Simmel FC, Pirzer T (2019). Periodic operation of a dynamic DNA origami structure utilizing the hydrophilic–hydrophobic phase-transition of stimulus-sensitive polypeptides. Small.

[78] Zadegan RM, Jepsen MDE, Thomsen KE, Okholm AH, Schaffert DH, Andersen ES, Birkedal V, Kjems J (2012). Construction of a 4 zeptoliters switchable 3D DNA box origami. ACS Nano.

[79] Hernández-Ainsa S, Keyser UF (2014). DNA origami nanopores: developments, challenges and perspectives. Nanoscale.

[80] Bell NAW, Keyser UF (2014). Nanopores formed by DNA origami: a review. FEBS Lett.

[81] Hernández-Ainsa S, Keyser UF (2013). DNA origami nanopores: an emerging tool in biomedicine. Nanomedicine (Lond).

[82] Li J, Stein D, McMullan C, Branton D, Aziz MJ, Golovchenko JA (2001). Ion-beam sculpting at nanometre length scales. Nature.

[83] Krishnan S, Ziegler D, Arnaut V, Martin TG, Kapsner K, Henneberg K, Bausch AR, Dietz H, Simmel FC (2016). Molecular transport through large-diameter DNA nanopores. Nat Commun.

[84] Raveendran M, Lee AJ, Sharma R, Wälti C, Actis P (2020). Rational design of DNA nanostructures for single molecule biosensing. Nat Commun.

[85] Song L, Jiang Q, Wang Z-G, Ding B (2017). Self-assembled DNA nanostructures for biomedical applications. ChemNanoMat.

[86] Shen B, Piskunen P, Nummelin S, Liu Q, Kostiainen MA, Linko V (2020). Advanced DNA nanopore technologies. ACS Appl Bio Mater.

[87] Hernández-Ainsa S, Bell NAW, Thacker VV, Göpfrich K, Misiunas K, Fuentes-Perez ME, Moreno-Herrero F, Keyser UF (2013). DNA origami nanopores for controlling DNA translocation. ACS Nano.

[88] Thomsen RP, Malle MG, Okholm AH, Krishnan S, Bohr SSR, Sørensen RS, Ries O, Vogel S, Simmel FC, Hatzakis NS, Kjems J (2019). A large size-selective DNA nanopore with sensing applications. Nat Commun.

[89] Mei Q, Wei X, Su F, Liu Y, Youngbull C, Johnson R, Lindsay S, Yan H, Meldrum D (2011). Stability of DNA origami nanoarrays in cell lysate. Nano Lett.

[90] Conway JW, McLaughlin CK, Castor KJ, Sleiman H (2013). DNA nanostructure serum stability: greater than the sum of its parts. Chem Commun.

[91] Zhang F, Jiang S, Wu S, Li Y, Mao C, Liu Y, Yan H (2015). Complex wireframe DNA origami nanostructures with multi-arm junction vertices. Nat Nanotechnol.

[92] Chen J, Seeman NC (1991). Synthesis from DNA of a molecule with the connectivity of a cube. Nature.

[93] Shih WM, Quispe JD, Joyce GF (2004). A 1.7-kilobase single-stranded DNA that folds into a nanoscale octahedron. Nature.

[94] Juul S, Iacovelli F, Falconi M, Kragh SL, Christensen B, Frøhlich R, Franch O, Kristoffersen EL, Stougaard M, Leong KW, Ho Y-P, Sørensen ES, Birkedal V, Desideri A, Knudsen BR (2013). Temperature-controlled encapsulation and release of an active enzyme in the cavity of a self-assembled DNA nanocage. ACS Nano.

[95] Liu Z, Li Y, Tian C, Mao C (2013). A smart DNA tetrahedron that isothermally assembles or dissociates in response to the solution pH value changes. Biomacromolecules.

[96] Yang Y, Endo M, Hidaka K, Sugiyama H (2012). Photo-controllable DNA origami nanostructures assembling into predesigned multiorientational patterns. J Am Chem Soc.

[97] Bhatia D, Surana S, Chakraborty S, Koushika SP, Krishnan Y (2011). A synthetic icosahedral DNA-based host–cargo complex for functional in vivo imaging. Nat Commun.

[98] Kim K-R, Lee Y-D, Lee T, Kim B-S, Kim S, Ahn D-R (2013). Sentinel lymph node imaging by a fluorescently labeled DNA tetrahedron. Biomaterials.

[99] Tapio K, Bald I (2020). The potential of DNA origami to build multifunctional materials. Multifunct Mater.

[100] Hui L, Xu A, Liu H (2019). DNA-based nanofabrication for antifouling applications. Langmuir.

[101] Thompson M, Blaszykowski C, Sheikh S, Rodriguez-Emmenegger C, Pereira A de los S (2016) Biological fluid-surface interactions in detection and medical devices. Royal Society of Chemistry, Cambridge.

[102] D’Agata R, Bellassai N, Giuffrida MC, Aura AM, Petri C, Kögler P, Vecchio G, Jonas U, Spoto G (2021). A new ultralow fouling surface for the analysis of human plasma samples with surface plasmon resonance. Talanta.

[103] Bellassai N, Marti A, Spoto G, Huskens J (2018). Low-fouling, mixed-charge poly-l-lysine polymers with anionic oligopeptide side-chains. J Mater Chem B.

[104] Bellassai N, Spoto G (2016). Biosensors for liquid biopsy: circulating nucleic acids to diagnose and treat cancer. Anal Bioanal Chem.

[105] Bellassai N, D’Agata R, Jungbluth V, Spoto G (2019). Surface plasmon resonance for biomarker detection: advances in non-invasive cancer diagnosis. Front Chem.

[106] D’Agata R, Bellassai N, Allegretti M, Rozzi A, Korom S, Manicardi A, Melucci E, Pescarmona E, Corradini R, Giacomini P, Spoto G (2020). Direct plasmonic detection of circulating RAS mutated DNA in colorectal cancer patients. Biosens Bioelectron.

[107] Daems D, Pfeifer W, Rutten I, Saccà B, Spasic D, Lammertyn J (2018). Three-dimensional DNA origami as programmable anchoring points for bioreceptors in fiber optic surface plasmon resonance biosensing. ACS Appl Mater Interfaces.

[108] Lubin AA, Plaxco KW (2010). Folding-based electrochemical biosensors: the case for responsive nucleic acid architectures. Acc Chem Res.

[109] Li G, Fu H, Chen X, Gong P, Chen G, Xia L, Wang H, You J, Wu Y (2016). Facile and sensitive fluorescence sensing of alkaline phosphatase activity with photoluminescent carbon dots based on inner filter effect. Anal Chem.

[110] He J, Hu X, Gao X, Meng C, Li Y, Li X, Fan L, Yu H-Z (2020). A versatile fluorometric in situ hybridization method for the quantitation of hairpin conformations in DNA self-assembled monolayers. Analyst.

[111] Liang M, Pan M, Hu J, Wang F, Liu X (2018). Electrochemical biosensor for microRNA detection based on cascade hybridization chain reaction. ChemElectroChem.

[112] Gao F, Du L, Tang D, Lu Y, Zhang Y, Zhang L (2015). A cascade signal amplification strategy for surface enhanced Raman spectroscopy detection of thrombin based on DNAzyme assistant DNA recycling and rolling circle amplification. Biosens Bioelectron.

[113] Kamal A, She Z, Sharma R, Kraatz H-B (2017). A study of the interactions of Hg(II) with T-T mispair containing hairpin loops. Electrochim Acta.

[114] Xu W, Zhao A, Zuo F, Jafar Hussain HM, Khan R (2019). A “turn-off” SERS aptasensor based DNAzyme-gold nanorod for ultrasensitive lead ion detection. Anal Chim Acta X.

[115] Xiong E, Wu L, Zhou J, Yu P, Zhang X, Chen J (2015). A ratiometric electrochemical biosensor for sensitive detection of Hg2+ based on thymine-Hg2+-thymine structure. Anal Chim Acta.

[116] Huang S, Feng M, Li J, Liu Y, Xiao Q (2018). Voltammetric determination of attomolar levels of a sequence derived from the genom of hepatitis B virus by using molecular beacon mediated circular strand displacement and rolling circle amplification. Microchim Acta.

[117] Petrovykh DY, Pérez-Dieste V, Opdahl A, Kimura-Suda H, Sullivan JM, Tarlov MJ, Himpsel FJ, Whitman LJ (2006). Nucleobase orientation and ordering in films of single-stranded DNA on gold. J Am Chem Soc.

[118] Huang J, Wu J, Li Z (2015). Biosensing using hairpin DNA probes. Rev Anal Chem.

[119] Keum J-W, Bermudez H (2009) Enhanced resistance of DNA nanostructures to enzymatic digestion. Chem Commun (Camb) 7036–7038 . 10.1039/b917661f. 10.1039/b917661f 19904386

[120] Soleymani L, Fang Z, Sargent EH, Kelley SO (2009). Programming the detection limits of biosensors through controlled nanostructuring. Nat Nanotechnol.

[121] Liu S, Su W, Li Z, Ding X (2015). Electrochemical detection of lung cancer specific microRNAs using 3D DNA origami nanostructures. Biosens Bioelectron.

[122] Monroy-Contreras R, Vaca L (2011). Molecular beacons: powerful tools for imaging RNA in living cells. J Nucleic Acids.

[123] Tay CY, Yuan L, Leong DT (2015). Nature-inspired DNA nanosensor for real-time in situ detection of mRNA in living cells. ACS Nano.

[124] Xie N, Huang J, Yang X, Yang Y, Quan K, Wang H, Ying L, Ou M, Wang K (2016). A DNA tetrahedron-based molecular beacon for tumor-related mRNA detection in living cells. Chem Commun.

[125] Corradini R (2018). Special issue: molecular properties and the applications of peptide nucleic acids. Molecules.

[126] Campbell MA, Wengel J (2011). Locked vs. unlocked nucleic acids (LNAvs.UNA): contrasting structures work towards common therapeutic goals. Chem Soc Rev.

[127] Pedersen RO, Kong J, Achim C, LaBean TH (2015). Comparative incorporation of PNA into DNA nanostructures. Molecules.

[128] Zhao Z, Fu J, Dhakal S, Johnson-Buck A, Liu M, Zhang T, Woodbury NW, Liu Y, Walter NG, Yan H (2016). Nanocaged enzymes with enhanced catalytic activity and increased stability against protease digestion. Nat Commun.

[129] Hahn J, Wickham SFJ, Shih WM, Perrault SD (2014). Addressing the instability of DNA nanostructures in tissue culture. ACS Nano.

[130] Xiao M, Lai W, Man T, Chang B, Li L, Chandrasekaran AR, et al. Rationally engineered nucleic acid architectures for biosensing applications. 2019. 10.1021/acs.chemrev.9b00121. 10.1021/acs.chemrev.9b00121 31573184

[131] D’Agata R, Spoto G (2019) Advanced methods for microRNA biosensing : a problem-solving perspective. Anal Bioanal Chem 1–20 . 10.1007/s00216-019-01621-8. 10.1007/s00216-019-01621-8 30710205

[132] Wang J, Zhang L, Lu L, Kang T (2019). Molecular beacon immobilized on graphene oxide for enzyme-free signal amplification in electrochemiluminescent determination of microRNA. Microchim Acta.

[133] Lee JH, Kim JA, Jeong S, Rhee WJ (2016). Simultaneous and multiplexed detection of exosome microRNAs using molecular beacons. Biosens Bioelectron.

[134] Cho S, Yang HC, Rhee WJ (2019). Simultaneous multiplexed detection of exosomal microRNAs and surface proteins for prostate cancer diagnosis. Biosens Bioelectron.

[135] Xu W, Yin P, Dai M (2018). Super-resolution geometric barcoding for multiplexed miRNA profiling. Angew Chemie - Int Ed.

[136] Schlichthaerle T, Strauss MT, Schueder F, Woehrstein JB, Jungmann R (2016). DNA nanotechnology and fluorescence applications. Curr Opin Biotechnol.

[137] Jungmann R, Avendaño MS, Woehrstein JB, Dai M, Shih WM, Yin P (2014). Multiplexed 3D cellular super-resolution imaging with DNA-PAINT and Exchange-PAINT. Nat Methods.

[138] Fang W, Jia S, Chao J, Wang L, Duan X, Liu H, Li Q, Zuo X, Wang L, Wang L, Liu N, Fan C (2019). Quantizing single-molecule surface-enhanced Raman scattering with DNA origami metamolecules. Sci Adv.

[139] Funck T, Nicoli F, Kuzyk A, Liedl T (2018). Sensing picomolar concentrations of RNA using switchable plasmonic chirality. Angew Chemie - Int Ed.

[140] Zanoli LM, D’Agata R, Spoto G (2012). Functionalized gold nanoparticles for ultrasensitive DNA detection. Anal Bioanal Chem.

[141] Puchkova A, Vietz C, Pibiri E, Wünsch B, Sanz Paz M, Acuna GP, Tinnefeld P (2015). DNA origami nanoantennas with over 5000-fold fluorescence enhancement and single-molecule detection at 25 μm. Nano Lett.

[142] Ochmann SE, Vietz C, Trofymchuk K, Acuna GP, Lalkens B, Tinnefeld P (2017). Optical nanoantenna for single molecule-based detection of Zika virus nucleic acids without molecular multiplication. Anal Chem.

[143] Adegoke O, Park EY (2016). Gold nanoparticle-quantum dot fluorescent nanohybrid: application for localized surface plasmon resonance-induced molecular beacon ultrasensitive DNA detection. Nanoscale Res Lett.

[144] Kielar C, Xin Y, Shen B, Kostiainen MA, Grundmeier G, Linko V, Keller A (2018). On the stability of DNA origami nanostructures in low-magnesium buffers. Angew Chemie Int Ed.

[145] Porchetta A, Ippodrino R, Marini B, Caruso A, Caccuri F, Ricci F (2018). Programmable nucleic acid nanoswitches for the rapid, single-step detection of antibodies in bodily fluids. J Am Chem Soc.

